# Comprehensive Numerical Analysis of Finite Difference Time Domain Methods for Improving Optical Waveguide Sensor Accuracy

**DOI:** 10.3390/s16040506

**Published:** 2016-04-09

**Authors:** M. Mosleh E. Abu Samak, A. Ashrif A. Bakar, Muhammad Kashif, Mohd Saiful Dzulkifly Zan

**Affiliations:** Department of Electrical, Electronic and Systems Engineering, Faculty of Engineering and Built Environment, University Kebangsaan Malaysia, 43600 UKM Bangi, Selangor, Malaysia; ashrif@eng.ukm.my (A.A.A.B.); kaashpk@bzu.edu.pk (M.K.); saiful@vlsi.eng.ukm.my (M.S.D.Z.)

**Keywords:** surface plasmon resonance SPR, FDTD methods, multi-dimensional FDTD, optical waveguide sensor OWS

## Abstract

This paper discusses numerical analysis methods for different geometrical features that have limited interval values for typically used sensor wavelengths. Compared with existing Finite Difference Time Domain (FDTD) methods, the alternating direction implicit (ADI)-FDTD method reduces the number of sub-steps by a factor of two to three, which represents a 33% time savings in each single run. The local one-dimensional (LOD)-FDTD method has similar numerical equation properties, which should be calculated as in the previous method. Generally, a small number of arithmetic processes, which result in a shorter simulation time, are desired. The alternating direction implicit technique can be considered a significant step forward for improving the efficiency of unconditionally stable FDTD schemes. This comparative study shows that the local one-dimensional method had minimum relative error ranges of less than 40% for analytical frequencies above 42.85 GHz, and the same accuracy was generated by both methods.

## 1. Introduction

The electrons on differently charged metal boundaries can exhibit clear coherent fluctuations that are confined to both boundary sides of an optical waveguide sensor (OWS). A surface plasmon resonance (SPR) wave is a wave that propagates along both metallic surfaces and in certain dielectric materials. A plasmon wave’s electric field reaches its peak value at the surface and decays evanescently away from the surface. The plasmon wave’s properties include high sensitivity to any changes in a device’s geometry or the refractive index of the material. As a full wave modeling method, FDTD is the most effective algorithm for modeling these types of devices. Furthermore, surface plasmons are increasingly popular due to the interaction between light and matter, which is controlled by patterned structures. Usually, the micro-integrated sensor normalizes the employed optical signals to the reference sensing channels based on the refractive index with accurate resolution and increased stability. The performance of the optical waveguide sensor elements can be verified using multi-dimensional finite difference time domain (FDTD) methods by employing specific arithmetic operations, relative errors and time stepping approaches. For the purpose of evaluating and improving the accuracy of optical waveguide sensors, generally, the achieved accuracy is not much better than that obtained using other FDTD methods. Furthermore, most of the difficulties in FDTD techniques have arisen because of the interfaces of the utilized material. Computational electromagnetics has been an important area of research because of its capability to accurately and flexibly model and simulate real processes in actual electrical and electronic circuits and systems. The various numbers used in (FDTD) techniques for modeling microwave propagation components have been recently illustrated and published. This paper demonstrates a comprehensive numerical analysis of several quantitative finite different time domain methods to determine which of the methods are most accurate for optimizing optical waveguide sensor resonance frequencies. The resonance frequencies were partially changed to model the physical medium’s properties with the best fit by using the most efficient numerical technique. Our objective was to analyze the reliability and accuracy of the FDTD methods. For completeness, the existing FDTD techniques for optical waveguides will be introduced and evaluated along with the three main methods: the Kane Yee-FDTD scheme, LOD-FDTD method, and ADI-FDTD method. The previous finite difference time domain methods based on finite domain FD direct approximations of the medium field equations are clearly illustrated in [1,2]. Those equations illustrate which of the used models are more responsive to the field’s current by using the auxiliary ordinary differential equations and Maxwell’s coupled equations. 

Generally, these FDTD techniques were referenced to Young’s direction implicit (DI) method. Other techniques were built on the Maxwell difference approximation equations, which were combined with the iteration convolution integral derived from the auxiliary differential equation, as clearly illustrated in [3,4]. The recursive convolution (RC) method, which is also called the convolution integral technique, has been used effectively in various applications such as magnetized cold plasmas [5] and dispersive dielectrics [6,7]. In addition, the transmission-line matrix (TLM) method and the Z-transform method were described in [8,9], respectively. An optical waveguide sensor is a physical sensing structure that detects guided electromagnetic (EM) waves in an optical wave spectrum detector. This definition includes rectangular integrated waveguides and cylindrical optical fiber sensors. Basically, the operating range of these sensor-based surface plasmon resonance (SPR) systems is defined by the operating wavelength and the materials’ refractive indices, and for conventional waveguide sensors, the operational range should be above 1.4. Moreover, the wavelengths should be visible and closed to the infrared region. 

The shifting requirements of the operating range of integrated optical waveguide sensors should include aqueous environments. SPR systems have been considered as high-efficiency detection techniques due to their high sensitivity and label-free capability [10] and have been chosen as a perfect example of optical waveguide sensors for comprehensive numerical analyses to improve the accuracy. Optical waveguide-based SPR sensors are commonly used to control the paths of optical rays (numerous attractive features) in small and rugged sensor systems, for example, to suppress the effects of stray rays and to efficiently control the properties of the rays. 

**Figure 1:**
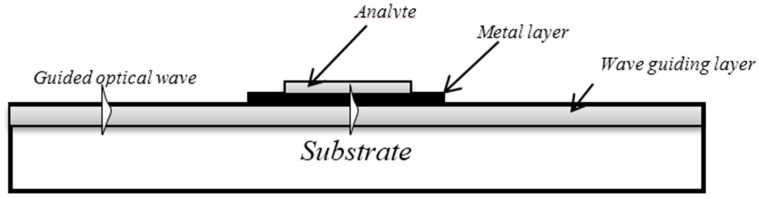
Optical waveguide-based SPR system.

Generally, in optical waveguide-based SPR systems, the reflected beam covers the same angle ranges when the beam emission forms the band and the specific range of the incident resonance angles is produced by a converging light. Theoretically, waveguide-based SPR sensitivity devices are equivalent to the corresponding ATR sensitivity configurations. The designing constraints were increased by using a specific, despite comparative, case for bulk prism-based SPR-sensing devices. Figure 1 shows an optical waveguide-based SPR system that includes three layers (analyte, metal layer, and waveguide layer) together with the optical waveguide.

### 1.1. Optical Planar Waveguide

The optical planar waveguide is a main element of photonic devices and performs all the optical signal operations, which can include guiding, coupling, switching, splitting, multiplexing and de-multiplexing. The planar waveguide components, electro-optical components, transmitters, receivers, and driving electronics can be integrated into a single chip using planar technology, similar to microelectronics. Although the operation of optical waveguide sensors and components has been researched and is perfectly understood, their particular performance relies on many parameters, including the geometry, wavelength, initial field distribution, material data, and electro-optic driving conditions. These parameters must be optimized before fabricating a device. For a large-scale optoelectronic circuit, accurate modeling is a predominant issue because of the numerous resources required to fabricate a chip. Optical planar waveguide design relies on simulating the propagation of light signals, waveguide modes, mode coupling, losses and gains. The 1st part of the entry data is used to configure numerical calculations. Ideally, entry systems hide or limit the details of the numerical calculations. The 2nd part of the entry data defines the waveguide device in terms of its geometry, fabrication parameters, and material constants. Using an effective simulator with a project layout that can also handle fabrication parameters is the best way to process waveguide data. However, because waveguide modeling often uses sophisticated numerical algorithms, you must be familiar with some aspects of the underlying numerics. Planar waveguides are the building blocks of photonic circuits. The width of a waveguide is defined perpendicular to the path of the waveguide centerline.

**Figure 2:**
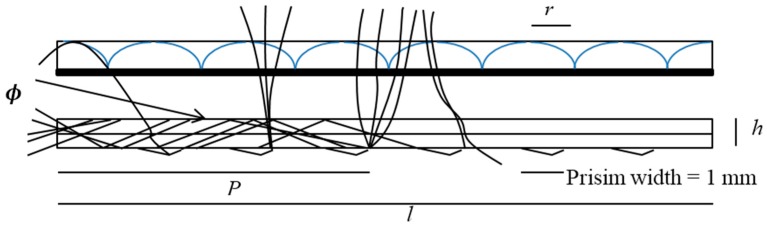
The optical waveguide’s parameters (Lateral section).

The numerical analysis of the optical waveguide sensor’s accuracy (the analytic models of the planar concentrator with cylindrical lenses) has been already derived by Karp. Accordingly, the geometrical concentration factor can be defined as the ratio of the thickness *h* to the length *l*. The analytic planar concentrator’s model can be applied to a single-axis planar concentrator [11]. The concentration parameter of the cylindrical lens is the main difference, which will be very small in that case. All the parameters were defined for various positions *P* and waveguide coupling angles *Φ*, which was integrated over all the propagation angles in the optical planar waveguide. In addition, the light coupling efficiency was defined for the waveguide coupling angles *Φ* along the waveguide at point *P*. As illustrated in Figure 2 and Equations (1)–(4), the commonly used Kretschmann’s prism beam configuration was applied to the metal film. Kretschmann *et al.* first demonstrated the attenuated total reflectance (ATR) technique. In principal, the SPW exciting process of an OWS is very similar to the Kretschmann ATR coupler process. The wave rays excite the SPW at the external metal’s interface when a guided mode and the SPW are phase matched. An optical waveguide guides the light waves that enter the region through the use of a thin overlayer of metal; the light evanescently penetrates the metal layer. This behavior can be depicted using the following equation:

(1) $$\eta_{decouple}\;\left(\rho ,\phi \right)=\;{\left(1-\frac{1}{C\mathrm{cyltnd}.\mathrm{lens}}\right)}^{P\tan\phi /2h}$$

The reflection coefficient of the coupling prism and the material absorption is calculated using:

(2) $$\eta_{decouple}\;\left(\rho ,\phi \right)=Ύ\times \;\eta_{\mathrm{decouple}}\;\times \;e^{\;\frac{-\alpha \;P}{cos\;\phi }}$$

The complete concentrator’s optical efficiency was derived by integrating over all the coupled angle points along the waveguide at point *P*, as in Equation (3):

(3) $$\eta_{total}=\;\frac{\sum_{P}\int_{0}^{\phi \;max}\eta_{position}\left(\;P,\;\phi \right)}{\left(l-r\right)/2r}$$

Figure 2 shows the parameters used to calculate the efficiency of the optical system. It shows that the light concentrates in the optical planar waveguide. 

*Ύ* is the metallized coupling prism’s reflection coefficient, r is the half pitch of the cylindrical lens, *α* is the attenuation coefficient of the waveguide material, *h* is the waveguide thickness, l is the waveguide length, and *P* is the distance to an exit surface (a specific position inside the waveguide). A cylindrical lens array is placed in the waveguide, and a coupling prism is placed at the central point of the lens for coupling sunlight into the waveguide. Accordingly, the light rays travel along the waveguide and exit on the waveguide’s side; the neglected integration limit was equal to 0, whereas the upper limit was *Φ_max_*, as illustrated in Equation (4) by using the same parameters as in the previous equation:

(4) $$\phi_{\max}=2\;(\;\theta +\tan^{-1}\left(\frac{1}{2nf/\theta }\right))$$

In the last equation, the variable (f/*θ*) is the ratio between the focal length and the diameter of the cylindrical lens, the constant n is the waveguide refractive index, and the angle *θ* is the half angular extension of the sun (± 0.26°). A substantial issue of Equation 1 requires careful note. This issue is the reliance of a ray’s coupling angle *Ф* on the optical efficiency of the optical waveguide. The ray’s coupling angle *Ф* impacts the value of (2HtanΦ), which represents the distance between double backside interactions. Accordingly, larger-angle coupled rays have a much greater impact along the backside surface, which will greatly increase the probability that a ray will escape the waveguide by striking the 2nd coupling prism during its propagation [12]. Usually a dark line will appear in the band when surface plasmon resonance occurs at specific spread angles [13]. 

Given the behavior of the rays as a function of the input wave vector, this structure will generate diffraction orders and act as a diffraction grating, and terms m= {…,−2,−1,0,1,2,…} will be generated in the reflected light. The optical solution technique based on the real time detection (RTD) method delivers unique results across diverse categories of related research. The resonance causes the light ray to be absorbed. The surface plasmon (SP) was set to resonate with the light when the light beam entered the metal film at a resonance angle. The tunability of the incoming wave vector to match the wave vector is an important prerequisite for surface plasmon (SP) excitation. Currently, kx can be set to any value between the line pairs labeled (*L1*) and (*L2*). One such line, labeled (*L3*), is shown. Lines (*L1*) and (*L2*) are the dispersion relations for “normal” light in the 2 mediums (ε_1_) and (ε_3_), and they both depend on the incidence angle α in the experimental setup shown in the inset. By varying the incidence angle α, any line (*L3*) between the two lines (*L1*) and (*L2*) can be achieved. 

### 1.2. Optical Rib Waveguide Array

**Figure 3:**
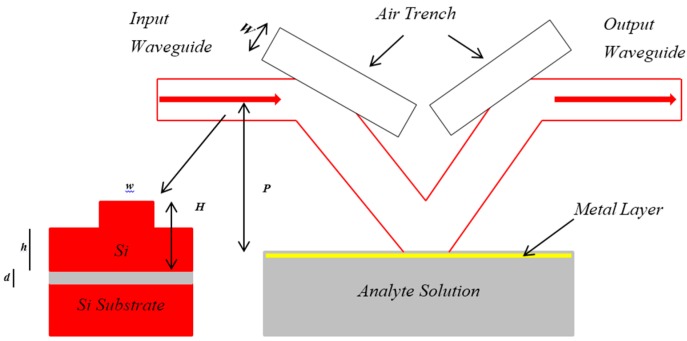
A view of the top of an optical rib waveguide array-based surface plasmon resonance sensor and the identical cross-section of a SOI rib waveguide.

A view of the top of the surface plasmon resonance (SPR) sensor and the identical cross-section of the silicon-on-insulator (SOI) rib waveguide are illustrated in Figure 3. It contains four components: the V-shaped bend waveguide, input/output waveguides, a double deeply etched facet coating with a metal layer (a surface plasmon resonance interface) and double air trenches. The third component is used to connect the first component with the second component; therefore, double bends in the silicon-on-insulator (SOI) rib waveguides are formed. Thus, the second component is in a straight line and parallel to the third component. 

*d* is the distance between the 3rd component and the centerline of the 2nd component, and the half-angle of the 1st component is *θ*. *L* and *W* represent the width and length of the fourth component, respectively. The surface plasmon resonance array’s sensor could be composed to implement the wavelength interrogation of the sensor’s output signal using the features of silicon micro-fabrication technology and the silicon-on-insulator rib waveguide. Accordingly, bulky and expensive equipment, such as spectrometers, are not necessary; a spectrometer optimizes the surface plasmon resonance array’s sensor for the accurate integration and miniaturization of the complete sensing system [14]. The surface plasmon resonance is completely excited by the guiding mode at the third component (the bend waveguide interface). Accordingly, a bimetallic configuration was employed. The two-dimensional surface plasmon resonance sensor can be simulated and numerically modeled using the 2D-FDTD method with perfectly matched layers (PMLs). The effective index method (EIM) is the fundamental technique for that purpose. The silicon-on-insulator (SOI) rib waveguide structure was numerically modeled as a two-dimensional structure. It is well known that FDTD modeling results in high accuracy and requires large amounts of computer memory and computational time. Basically, the surface plasmon resonance excitation mechanism of a surface plasmon resonance sensor based on a silicon-on-insulator rib waveguide is similar to that of the Kretschmann prism geometry (classical surface plasmon resonance model); the improvement in the resonance condition is illustrated by the following equations:

(5) r ≥ 0.5

(6) $$t\;<\;c\;+\;\frac{r}{\sqrt{1-r^{2}}}$$

(7) k0= 2πλ0 

(8) $$k_{0}\;n_{\mathrm{eff}}\;\sin\theta =K_{\mathrm{SPR}}=k_{0}\sqrt{\frac{\epsilon_{m}\;\epsilon_{d}\;}{\epsilon_{m}+\epsilon_{d}}}$$

where $\lambda_{0}$ is the optical wavelength in free space, ε_m_ is the dielectric constant of the metal solution, ε_d_ is the dielectric constant of the analyte solution, and n_eff_ is the effective refractive index of the guiding mode of the silicon-on-insulator rib waveguide. Finally, the surface plasmon resonance sensor (SPR) based on a silicon-on-insulator (SOI) rib waveguide is categorized as a special model of Kretschmann that can include a p-polarized incident beam evolved from the TE-polarized guiding mode of the silicon-on-insulator (SOI) rib waveguide and a virtual prism whose refractive index is equal to the effective refractive index of the guiding mode. 

## 2. Basic FDTD Methods

**Figure 4:**
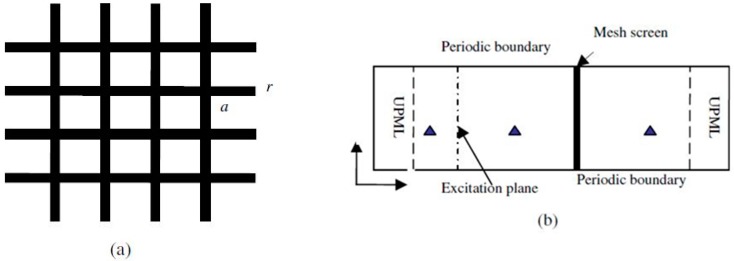
Two major models of wire mesh screens. (**a**) The physical model. (**b**) The numerical model.

The fundamental unification of the electric and magnetic field equations is represented by the partial differential equations for waveguide micro-components. Usually, these equations benchmark the necessary reference points for optimizing the optical waveguide sensor accuracy, in addition to the specific fundamentals, theories, and principles such as sensitivity, signal-to-noise ratio, precision, induction, repeatability, duality, and minimum detection limit. The approximate solutions of these equations are under wide investigation for analyses of optical waveguide sensor issues, radiation effects, and scattering characteristics. Various numerical approaches are available for the modeling, simulation, and computation of wave propagation in optical waveguides. The 1st methods, recursive convolution (RC), are composed of hybrid particle-fluid (HPF) methods, kinetic particle simulations, and magneto hydrodynamics. The second method, direction implicit (DI), basically diagnoses the problems of nonlinear high-power wave propagation via the coupled Maxwell’s equations, which are specific for the Boltzmann electron velocity distribution function equations. Moreover, both methods are completely convenient to use with varied physical requirements. Given the physical conditions of an optical waveguide sensor, the low-level characterization of the wave propagation power has been perfectly simulated using magneto-ionic (MI) theory; MI theory neglects the thermal velocity value, which is close to zero for the charged species. Furthermore, TLM and Z-transform methods have been described in [5,6], respectively. When reduced to the optical sensor case, the second method requires five state variables for the iteration process, which will result from the use of 6 state variables. In conclusion, previous methods are more difficult to analyze and are more computationally intensive than the direction implicit (DI) and recursive convolution (RC) methods, neither of which requires more than three state variables. Therefore, we will not illustrate both the TLM method and the Z-transform method here. Figure 4 illustrates two types of wire mesh screens: the physical model and the numerical model. The uniaxial perfectly matched layer (UPML) was divided by the mesh screen, which caused an excitation plane along the periodic boundary. 

The electrons on the differentially charged metal boundaries can exhibit clear coherent fluctuations that are confined to the both boundary sides of the optical waveguide sensor (OWS) surface. SPR is an OWS technique that agitates the deeply excavated OWS bent surface via a bimetallic configuration and the guiding mode. The surface charge induces the En field’s discontinuity along the surface normal z-axis, which causes the p-shape character of the surface plasmon waves; these waves do not have E_z_ or E_x_ components, only E_y_ [13].

### 2.1. Recursive Convolution (RC) Method

Generally, this method is an accurate second-order technique for analyzing an optical waveguide sensor (OWS) case. Moreover, another method, the total residual chlorine (TRC) method [7], has already been developed and applied to many optical sensor applications. This method is illustrated in [3] for a second-order Lorentz dielectric. Furthermore, an improved piecewise linear recursive convolution (PLRC) method has been developed and applied to dispersive dielectrics [4]. The improved method can be easily applied to optical waveguide sensors. The capability functions of the time-domain (TD) approach from [3] allowed the analysis of the prerequisite differential equation’s parameters, coefficients, and arithmetic operations, and the formulas presented in [4], which form a solid basis for the PLRC and TRC methods, have much greater accuracy, efficiency, and precision than the conventional recursive convolution method [8]. Similarly, numerical experiments have illustrated that the conventional recursive convolution method has far lower accuracy, efficiency, and precision than the other methods mentioned herein. Therefore, we will consider the PLRC method in this analysis. For the piecewise linear recursive convolution technique, (E^n^) usually has piecewise linear functional dependence per time period (Δt). E^n^/Δt is constant. The updated equation of the electrical field in the metal can be expressed as:

(9) $$E^{n+1}=\frac{\varepsilon_{\infty }-\xi^{0}}{\varepsilon_{\infty }+x^{0}-\xi^{0}}E^{n}+\frac{1}{\varepsilon_{\infty }+x^{0}-\xi^{0}}\phi^{n}+\frac{\Delta t/\varepsilon_{0}}{\varepsilon_{\infty }+x^{0}-\xi^{0}}\nabla x\;H^{n+1}$$

where:

Φ^n^ = (Δx° − Δξ°) E^n^ + Δξ° E^n−1^ + e ^–vc Δt^ ϕ^n−1^

$$x{}^{\circ}=\frac{\omega_{p^{2}}}{v_{c}}[\Delta t-\frac{1}{v_{c}}(1-e^{-vc\;\Delta t})]$$

$$\Delta x{}^{\circ}=\frac{\omega_{p^{2}}}{v_{c^{2}}}{\left(1-e^{-vc\;\Delta t}\right)}^{2}$$

$$\xi_{0}=\frac{\omega_{p^{2}}}{v_{c}}[\frac{\Delta t}{2}-\frac{1}{v_{c^{2}}\Delta t}(1-e^{-vc\;\Delta t})+\frac{1}{v_{c}}e^{-vc\;\Delta t}]$$

$$\Delta \xi_{0}=\frac{\omega_{p^{2}}}{v_{c^{2}}}[\frac{1}{v_{c}\Delta t}{\left(1-e^{-vc\;\Delta t}\right)}^{2}+(1-e^{-vc\;\Delta t})e^{-vc\;\Delta t}]$$

### 2.2. Direction Implicit (DI) Method 

The Young’s direction implicit method is based on several approximations, including substituting ${ℐ}^{n+1/2}$ for ℐ(t) in the 2nd Maxwell equation; from Maxwell’s equations:

(10) $$\frac{\partial ℐ}{\partial \;t}+v\;J=\epsilon_{0}\;{\omega_{P}^{2}}_{E}$$

where (v) and (w_p_) are the electron-natural collision frequency and photon frequency, respectively. A completely equivalent equation form was used in [2], which led to the following difference equation:

(11) $$\frac{J^{n+1/2}-{ℐ}^{n-1/2}}{\Delta t}\;+v\frac{J^{n+1/2}-{ℐ}^{n-1/2}}{2}\;=\epsilon_{0}\;\omega_{P}^{2}E_{n}$$

Young’s direction implicit method requires that the electrical field component (E^n^) is stored at only a single time. Young’s DI method uses very few multiple components, but it uses more operations/Δt than any of the other methods, as illustrated in Nickisch- Franke’s direction implicit (NFDI) method [2]. This method is slightly different from the original method because the term $\frac{J^{n+1}+J^{n}}{2}$ was included for J(t):

(12) $$\frac{J^{n+1}-J^{n-1}}{2\Delta t}+v\;J^{n}=\epsilon_{0}\;{\omega_{P}^{2}}_{E^{n}}$$

Equations (10) and (11) are accurate second order approximations [15]. Furthermore, the storage requirements and capacities for the NFDI method are larger than those for the original DI method. The derivation of the RC method is completely different; for double (ℐ) time levels, it might be fully stored, but the iteration of the result requires a coupled set of first, second and third order differential equations, as in the Young’s direction implicit case. The new technique was based on the integral relating the time domain, namely, (D) and (E), rather than on the ordinary relations of the differential equation, namely, (ℐ) and (E). 

The included comparisons are quite similar to the first order dispersive dielectric FDTD method in the Steven direction implicit method, which has already been derived in [16]. The NFDI method, which was called the new direction implicit technique, including the term (${ℐ}^{n+1}+\;{ℐ}^{n}\;)\;/2$ for ℐ(t), can finally be presented:

(13) $$\frac{J^{n+1}-{ℐ}^{n}}{\Delta t}+v\frac{J^{n+1}+{ℐ}^{n}}{2}=\epsilon_{0}\;\omega_{P}^{2}\frac{E^{n+1}+E^{n}}{2}$$

## 3. Improved FDTD Methods

Finite difference time domain methods are approximate approaches for solving Maxwell’s partial differential equations. They have been widely used for solving a wide range of EM wave problems. The method can be applied to electromagnetic (EM) problems with different boundary shapes, different types of boundary conditions, and regions that contain a number of different materials. The application of the FDM is simple and straightforward because it involves only simple arithmetic for the derivation of the discretized equations and for writing the corresponding computer codes. From 1950–1970, the finite difference method (FDM) was the most important numerical method for solving practical EM problems. With the development of high-speed computers with large-scale storage capacities and the ease with which the finite difference method was applied, the method became increasingly popular for solving electromagnetic problems. 

FDTD methods have become one of the widely and famously used schemes and will be discussed later on. Multiple microwave transmission lines normally utilize air-filled optical waveguides. Naturally, frequency domain methods can be computationally more efficient because only a narrow range of frequencies is of interest. However, they cannot easily address nonlinear elements. Time domain methods can provide solutions for wideband frequency spectra and can easily handle nonlinear elements, but they require a large number of iterations if a high frequency is of interest. The Fourier transform can be used to convert the results between the frequency and time domains. 

### 3.1. Alternating Direction Implicit (ADI) Method

The principle of this method that has been used for solving parabolic differential equations is utilized. However, a modified alternating direction implicit technique is developed and compared to the typical ADI techniques. The mixed coordinates are altered at each time step rather than each coordinate at every time step. Consequently, in the 3D case, the requirement is simply two changes in the solution identification. Therefore, the Courant-Fredericks-Levy (CFL) stability constraint is completely mitigated by the finite difference time domain methods, resulting in the modified ADI algorithm. The CFL stability conditions do not limit the FDTD time step as the modeling precision of the FDTD methods because of the mixing between the ADI and FDTD techniques. The ADI-FDTD approach is unconditionally stable based on a theoretical proof, and this proposed approach is efficient and effective, as predicted by numerical results. For a similar numerical precision, the proposed ADI-FDTD requires at least four times less numerical iteration than that of a typical FDTD method. The FDTD technique is very significant in electrical engineering. Eventually, the number of iterations and CPU time were decreased [15]. Nevertheless, the CFL stability constraint and numerical dispersion errors restrict the high Q structures and geometrical efficiency. The ADI-FDTD approach was developed to mitigate the Courant-Fredericks-Levy stability constraint. The error minimized ADI-FDTD and enhanced ADI-FDTD techniques were created to enhance the accuracy. Two approaches, PSTD and MRTD, were employed to minimize the numerical dispersion errors. Recently, the LOD-FDTD approach has been discussed as an unconditionally stable method, along with the CN-FDTD approach. The Crank-Nicolson method is computationally expensive but is more exact than the ADI-FDTD approach. Since Yee created the FDTD method, it has been successfully employed for a large number of problems. In addition, the CFL stability condition reduced the computational efficiency of FDTD, including for high Q structures in particular or structures where field variations occur rapidly. Therefore, the unconditionally stable CN-FDTD and ADI-FDTD approaches were proposed to address that restriction. Recently, the popularity of both techniques has increased because of unconditional stability. However, although ADI-FDTD is computationally effective, it has large errors when large time steps are used. This drawback of ADI-FDTD with unconditional stability compromises its utilization. However, high performance can be achieved using the CN-FDTD approach with large time steps, but it requires a larger computational period. Eventually, the benefits of both approaches are needed. In fact, from advance studies of both methods, it can be assumed that ADI-FDTD is an altered form of the CN-FDTD approach, which is known as the splitting error term. Accordingly, antagonistic techniques that can solve the Crank-Nicolson method in an alternating direction implicit platform have been illustrated. These techniques incorporate an iteration loop for each advance of Δt in the FDTD method. The ADI-FDTD algorithm [15] in the “Novel unconditionally stable FDTD method for electromagnetic and microwave modeling” developed by Zheng is numerically stable, even beyond the CFL point of the conventional FDTD. The original ADI technique has been applied to solve the parabolic differential equation and has proven to be stable. The ADI technique applied to the existing ADI-FDTD is unlike the original one. Rather than alternating the coordinate directions, from which ADI gets its name, the modified ADI technique used in ADI-FDTD is applied by alternating the order of the expressions on the right-hand side of the equations. Hence, the stability of the existing approach must be rigorously examined to demonstrate that the existing scheme is stable without any conditions. Preliminary numerical experiments show the stability of the existing ADI-FDTD even for time steps that are hundreds of times the threshold of the conventional FDTD. The conventional FDTD approach was developed by Yee [17]. The CFL stability condition limits the time step of the FDTD method, which is a marching-in-time method. Consequently, in some applications, the FDTD may require a large number of iterations when small time steps are needed, e.g., structures with fine geometries such as small via. The existing ADI-FDTD scheme does not require the CFL stability condition. The CFL condition does not restrict the time step but, rather, the accuracy of the modeling results and the Nyquist sampling limit. Hence, the number of iterations can be significantly decreased compared to the conventional FDTD when simulating the same structure. It will be shown that ADI-FDTD has an advantage in this case. Similar to any numerical method, numerical dispersion is inherent in the FDTD scheme. It is the result of the spatial discretization where the phase velocity of the numerical models in the FDTD is forced to change with the grid size, time step, wave propagation direction, and modal wavelength. These nonphysical results are produced by numerical dispersion, for example, pulse distortion and artificial anisotropy. To estimate the errors caused by numerical dispersion, the numerical dispersion relation of discrete lattices must be studied. For the conventional FDTD scheme, the numerical dispersion has been investigated extensively. In the homogenous waveguide case, the air-filled cavity has a dimension of 9 mm × 6 mm × 15 mm. For the ADI-FDTD and Yee’s FDTD approaches, a network with Δ L = 0.6 mm was used, resulting in 15 × 10 × 25 grid points. Note that that the time step Δt FDTD = 0.8 ps and the larger time step Δt i = Δt FDTD = 2.4 ps were chosen for FDTD and ADI-FDTD, respectively. The errors in both methods are comparable in Table 1, but the suggested ADI-FDTD yielded a 1.55× savings in CPU time.

In the inhomogeneous waveguide case, half of the cavity is occupied by a dielectric material with a relative permittivity of ε_r_ = 64, and the other half is occupied by air. The cavity has a dimension of l m × 2 m × 1.5 m. Again, a uniform mesh with ΔL = 0.1 m was used, which produced 10 × 20 × 15 mesh grid points, and a Δt FDTD = 1.5 × 10^−1^ s was chosen with the typical FDTD, whereas a higher time step of Δt i = 4 × Δt FDTD = 6 × 10^−10^ s was selected with the suggested ADI-FDTD. To establish the strength of the ADI-FDTD approach, one homogeneous and one inhomogeneous optical waveguide cavities were determined using the ADI-FDTD and the typical Yee’s FDTD. The cavities were chosen because analytical solutions are readily available for comparison in Table 2.

### 3.2. Locally One-Dimensional (LOD) Technique

It is very desirable to create unconditionally stable FDTD approaches that are free from the condition of CFL [18]. The celebrated alternating direction implicit (ADI) method is one of the methods based on FDTD. Recently, several scientists have suggested other unconditionally stable (non-dissipative) approaches such as split-step (SS) [19,20] and 1-D LOD-FDTD approaches [21,22]. LOD–FDTD techniques are used to establish 2D approaches related to the split-step process [19], which is designated as SS1. As normally contributed by scientists, LOD–FDTD produces an additional non-commutative error expression, consequently producing techniques precise to only the 1st stage in time.

For acquiring 2nd stage progressive accuracy, the Strang splitting technique can be implemented following the split-step technique [19], which is designated as SS2. Nevertheless, this technique requires extra arithmetic functions for executing three processes at every time period when calculating the fields, [20] and separate or parallel processes cannot be used in this case. It has been demonstrated that the substitute LOD–FDTD technique with the unconditionally stable property cannot produce the above-mentioned second stage commutative error. The three-dimensional Maxwell’s equation model can be presented as an LOD–FDTD. A numerical explanation will be given to establish its 2nd stage sequential accuracy. Moreover, the current three-dimensional LOD–FDTD approach includes two upgraded procedures that simply support the utilization of parallel and minimized outcome processing. The computational efficiency is higher than those that can be attained using the ADI-FDTD and SS2-FDTD approaches.

The LOD-FDTD approach is an additional example of a 2-D case; it is a two-stage technique, although its formulation is complex. It was observed that the previous approach was comparable to the split-step technique. Open structure problems can be resolved by convolutional perfectly matched layers (CPML), and perfectly matched layers (PML) have been effective for this approach, as shown by Iftikhar Ahmed *et al.* In the end, this research favors previous FDTD methods rather than the LOD-FDTD method, which includes three phases and is certainly well-defined by the common LOD method in the three spatial coordinate directions. The LOD approach can compute the equations, and the same number of equations can be computed using the typical ADI-FDTD approach, which requires more arithmetic operations. Table 3 compares both methods in term of arithmetic Operation Counts.

Therefore, LOD is more computationally efficient than the ADI-FDTD approach. The results were demonstrated based on the numerical calculations for the comparison of the analytical and typical ADI-FDTD approaches to demonstrate the usefulness of the existing approach. For validation purposes, the existing approach was employed on a 9 × 6 × 15 mm^3^ cavity. The selected cell sizes were Δx = Δy = Δz = 0.6 mm. The multiple modes had resonant frequencies that were computed and are shown in the following tables, and the analytical solutions and the solutions obtained with the typical ADI-FDTD approaches were compared in Table 4. It can be observed that ADI-FDTD and the current LOD-FDTD had similar relative errors. 

In this case, there were eight thousand iterations. Furthermore, with respect to computational cost, the two approaches were dissimilar; the LOD approach required a simulation time of 20 s and 1.336 MB of memory, but the ADI-FDTD approach required 25 s and 1.336 MB of memory. Explicitly, both approaches used the same amount of memory; however, LOD-FDTD was almost 20% faster than the ADI-FDTD approach. The simulation was performed on a Dell Pentium VI computer with 256 MB of RAM, and the program was created in C++ by Ahmed *et al.* the results of that simulation in terms of time steps, CPU time, and memory size are illustrated in Table 5.

## 4. Improving the Optical Waveguide Sensor’s Accuracy

The electromagnetic wave relations can be modeled by a FDTD approach commonly used for complex PCB structures. The conventional FDTD method that depends on Yee’s formulation is considered in this paper. The FDTD formulation, based on mathematical calculations and theorems and considering problems such as accuracy, computational complexity, stability, convergence, and dispersion, was easily described and derived by Yee. A three-dimensional model can be taken as a group of cubes that are known as Yee’s Cells in the FDTD approach. In this paper, all the cubes are taken as being similar in size, although cubes can have different sizes. The components of the magnetic and electric fields were modified in discrete time steps at an interval of 0.5Δt, where Δt is the time step. FDTD methods were extensively utilized to examine the plasmonic structures of the sensors based on the optical waveguide, and their applications for examining the SPR setups were restricted to a small number of typical Kretschmann configuration cases. Specifically, the wavelength output of the SPR setups based on the optical waveguide was not inspected using the FDTD methods, although the wavelength output response could be attained by applying the pulse excitation method based on the one-time solution. An FDTD analysis can be implemented even for small incident angles θ, but BPM cannot be utilized in this case. The previous facts can be a strong and exciting reason to examine the (FD) 2TD application. 

The primary numerical calculation issue for the electric field (E^n^) of an optical waveguide sensor with air filling regards the allowance factors, which has not yet been solved and is a real electromagnetic problem [23]. The electric field can be defined as:

*∇*^2^ φ = ρ/( ϵ ϵ_0_) (14)

By transforming $\nabla^{2}$ φ in the above equation into its equivalent:

(15) $$\frac{\delta }{\delta x}(\epsilon \;\frac{\delta \varphi }{\delta x})+\frac{\delta }{\delta y}(\epsilon \;\frac{\delta \varphi }{\delta y})+\frac{\delta }{\delta z}(\epsilon \;\frac{\delta \varphi }{\delta z})=-\rho /\left(\epsilon \right)$$

When ε=ε(E), Equation (15) becomes:

(16) $$\frac{\delta }{\delta x}(\epsilon \left(E\right)\frac{\delta \phi }{\delta x})+\frac{\delta }{\delta y}(\epsilon \left(E\right)\frac{\delta \phi }{\delta y})+\frac{\delta }{\delta z}(\epsilon \left(E\right)\frac{\delta \phi }{\delta z})=-\rho /\left(\epsilon \right)$$

It is very important to note that when the electrical field is strong, as in this case, the behavior of the medium will be nonlinear. The dependence between the electric field intensity E and the relative dielectric penetrability E then will also be nonlinear [24]:

(17) ϵ = 1.8 + 1.066 × 10^−5^ E

Substituting Equations (14) into (15), we obtain:

(18) $$\frac{\delta }{\delta x}(1.8+1.066\times 10^{-5}E\left(E\right)\frac{\delta \phi }{\delta x})+\frac{\delta }{\delta y}(1.8+1.066\times 10^{-5}E\left(E\right)\frac{\delta \phi }{\delta y})+\frac{\delta }{\delta z}(1.8+1.066\times 10^{-5}E\left(E\right)\frac{\delta \phi }{\delta z})=-\rho /\left(\epsilon \right)$$

**Figure 5:**
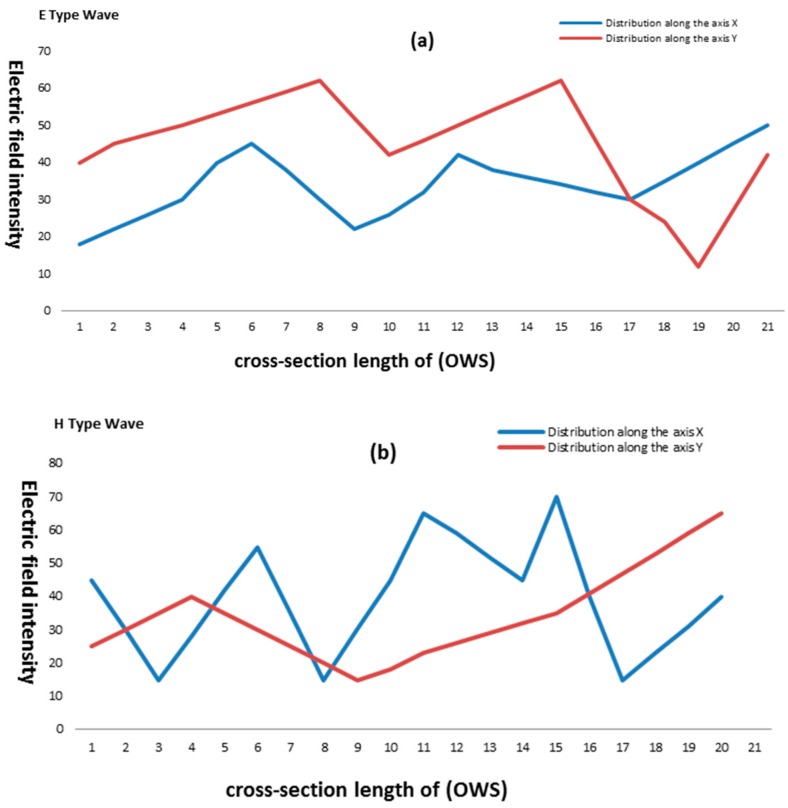
Dependencies between the electric field intensity and cross-section length of an OWS with air filling for E-type (**a**) and H-type (**b**) waves in the OWS.

Obtaining partial differential Equation (18) using experimental electric field models of an optical waveguide sensor with air filling was illustrated in Figure 5. Furthermore, the nonlinear and complex mathematical model was shown to be sufficiently full and precisely reflected the properties of the real system. Therefore, it is not necessary to obtain the general analytical solution of Equation (18) as long as we can solve it numerically.

The finite difference technique [25,26] is an efficient numerical technique, and consequently, it is the most commonly applied technique for solving partial differential equation initial-boundary value problems. The finite difference technique provides solutions based on a set of values demonstrating the field functions at certain discrete points spread uniformly over the entire area of the field. These values can be obtained by incorporating the partial differential equations of the demonstrated fields using an arrangement of ordinary FDTD equations that have linear equations that connect the potential values at one step with the potential values at another step in a closed loop. Therefore, to explain the field in the sensor based on an air-filled optical waveguide, it is 1st necessary to change differential Equation (18) into a group of linear algebraic equations and then solve the acquired system.

The main parameter that has been used is one of the optical waveguide cavity parameters, which makes the analytical solutions easier to read for the comparison of methods (Yee’s FDTD & ADI-FDTD in a Homogeneous/Inhomogeneous Air-filled Cavity). 

The main parameter that has been targeted for optimization is the resonant frequencies of an optical waveguide air-filled cavity that has the dimensions of 9 mm × 6 mm × 15 mm; a mesh with AI = 0.6 mm was used, resulting in 15 × 10 × 25 grid points. It can be seen that the errors for all the methods were comparable. However, a savings factor of 1.55 in CPU time was achieved with the proposed FDTD method in the analyzed case.

The material interfaces produced complications in FDTD while resolving the Maxwell’s equations solutions. Multiple types of problems arise if the geometrical parameters are much larger or smaller than a certain wavelength. In the former scenario, the core difficulty concerns the spatial discretization, which is why an improved geometrical flexibility with a comparatively higher accuracy is required. 

**Figure 6:**
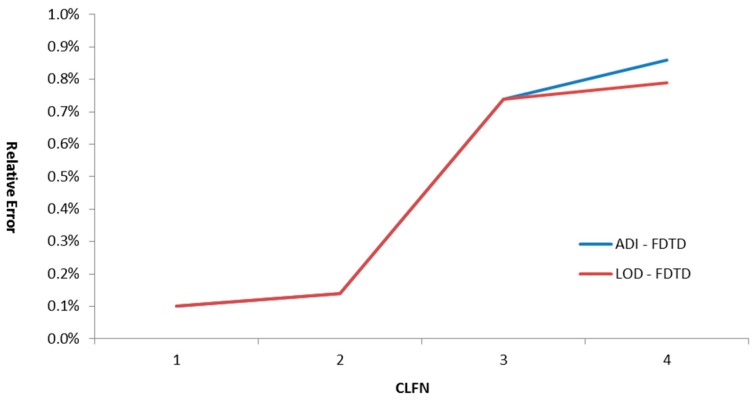
Comparative accuracy with respect to CFLN for an OWS.

Figure 6 shows the comparative accuracy (relative error) of the methods under comparison with respect to the CFL number. CFLN was defined as the ratio of the utilized time step to the Courant-Fredericks-Levy time limit for Yee’s scheme. 

$CFL=\;\frac{|u|\;\Delta t}{\Delta x\;}\leq 1$ for the one-dimensional convection equation.

From Figure 6, it is clear that both methods had the same relative errors during the first two intervals. Furthermore, the LOD-FDTD method was less computationally intensive; therefore, it is more computationally time efficient [27,28].

## 5. Conclusions

A three-dimensional ADI-FDTD technique developed by Zheng was used to optimize the resonance frequencies in an optical waveguide sensor. Yee’s scheme was used with the ADI application technique to edit part of the algorithm. As an outcome, the memory requirements of this method are approximately twice those of Yee’s scheme. Furthermore, the time-step is not highly restricted via the numerical stability conditions. Compared with earlier work reported in reference [16], the ADI-FDTD method minimizes the number of substeps by a factor of 2–3, which is a 33% time savings in a single round. The utilized numerical process demonstrated the accuracy of the LOD-FDTD technique without the CFL condition. Other researchers have preliminarily observed that the LOD-FDTD technique decreases the number of iteration by a factor of 0.25 for the same accuracy, which will decrease the iteration time by a factor of 1.6 over the conventional method. Note that parts of this manuscript were derived by Zheng. The comparative study showed that the LOD-FDTD technique had minimum relative error ranges that are > 40% for analytical frequencies above 42.85 GHz and had the same radio frequencies (RF) for alternative frequencies (AF) below that. The three steps of the unconditionally stable three-dimensional model were analytically compared using the ADI-FDTD technique. The alternating direction implicit technique can be considered as a significant step forward for improving the efficiency of unconditionally stable FDTD schemes. On the other hand, both methods have the same number of equations to be analyzed. Furthermore, the comparative study showed that the LOD-FDTD technique was approximately 20% faster than the alternating direction implicit technique. Furthermore, the two techniques had similar accuracies. 

## Figures and Tables

**Table 1:** Comparison of the homogeneous waveguide results of the Yee FDTD and ADI-FDTD methods.

Mathematical Results (GHz)	ADI–FDTD		Kane Yee Scheme	
	Simulation Results (GHz)	Relative Error	Simulation Results (GHz)	Relative Error
19.4270	19.4000	0.14%	19.4510	0.12%
26.0220	25.6910	0.23%	25.9720	0.19%
31.6520	31.5330	0.31%	31.4550	0.62%
34.7760	34.5770	0.57%	34.6130	0.47%

**Table 2:** Comparison of the inhomogeneous waveguide results of the Yee scheme and ADI-FDTD.

Mathematical Results (GHz)	ADI–FDTD		Kane Yee Scheme	
	Simulation Results (GHz)	Relative Error	Simulation Results (GHz)	Relative Error
18.6270	18.5870	0.21%	18.6100	0.11%
27.1720	27.0460	0.46%	27.1200	0.19%
29.3740	29.1550	0.88%	29.2250	0.51%
32.8810	32.6260	0.77%	32.6710	0.64%
35.0690	34.8320	0.67%	34.9460	0.35%

**Table 3:** Comparison of 3D LOD-FDTD and ADI-FDTD in terms of Arithmetic Operation Counts.

Arithmetic Operations		ADI-FDTD	LOD-FDTD
Implicit	M/D	18	12
	A/S	48	30
Explicit	M/D	12	6
	A/S	24	24
Total	M/D	30	18
	A/S	72	54

**Table 4:** Comparison of the results of the ADI-FDTD and 3D LOD-FDTD methods.

Analytical GHz	ADI-FDTD		LOD-FDTD	
	GHz	Relative Error	GHz	Relative Error
19.43	19.45	0.10%	19.45	0.10%
25.00	25.01	0.04%	25.01	0.04%
31.66	31.47	0.60%	31.47	0.60%
42.85	42.80	0.12%	42.87	0.05%

**Table 5:** Time step modeling and computational effort comparison.

	Δt	Steps	CPU Time	Memory
Kane Yee Scheme	0.96225 ps	2400	607.90 s	9.55 Mb
ADI-FDTD	0.96225 ps	2400	5488.3 s	23.6 Mb
LOD-FDTD	0.96225 ps	2400	5232.1 s	23.6 Mb
	9.62250 ps	240	524.10 s	23.6 Mb
	14.4338 ps	160	348.80 s	23.6 Mb
